# Specific Phenotypic Traits of Starmerella bacillaris Related to Nitrogen Source Consumption and Central Carbon Metabolite Production during Wine Fermentation

**DOI:** 10.1128/AEM.00797-18

**Published:** 2018-08-01

**Authors:** Vasileios Englezos, Luca Cocolin, Kalliopi Rantsiou, Anne Ortiz-Julien, Audrey Bloem, Sylvie Dequin, Carole Camarasa

**Affiliations:** a University of Torino, Dipartimento di Scienze Agrarie, Forestali e Alimentari, Agricultural Microbiology and Food Technology Sector, Grugliasco, Turin, Italy; b Lallemand SAS, Blagnac, France; c SPO, INRA, SupAgro, Université de Montpellier, Montpellier, France; The Pennsylvania State University

**Keywords:** *Starmerella bacillaris*, wine fermentation, carbon metabolism, nitrogen metabolism

## Abstract

Over the last few years, the potential of non-Saccharomyces yeasts to improve the sensory quality of wine has been well recognized. In particular, the use of Starmerella bacillaris in mixed fermentations with Saccharomyces cerevisiae was reported as an appropriate way to enhance glycerol formation and reduce ethanol production. However, during sequential fermentation, many factors, such as the inoculation timing, strain combination, and physical and biochemical interactions, can affect yeast growth, the fermentation process, and/or metabolite synthesis. Among them, the availability of yeast-assimilable nitrogen (YAN), due to its role in the control of growth and fermentation, has been identified as a key parameter. Consequently, a comprehensive understanding of the metabolic specificities and the nitrogen requirements would be valuable to better exploit the potential of Starm. bacillaris during wine fermentation. In this study, marked differences in the consumption of the total and individual nitrogen sources were registered between the two species, while the two Starm. bacillaris strains generally behaved uniformly. Starm. bacillaris strains are differentiated by their preferential uptake of ammonium compared with amino acids that are poorly assimilated or even produced (alanine). Otherwise, the non-Saccharomyces yeast exhibits low activity through the acetaldehyde pathway, which triggers an important redistribution of fluxes through the central carbon metabolic network. In particular, the formation of metabolites deriving from the two glycolytic intermediates glyceraldehyde-3-phosphate and pyruvate is substantially increased during fermentations by Starm. bacillaris. This knowledge will be useful to better control the fermentation process in mixed fermentation with Starm. bacillaris and S. cerevisiae.

**IMPORTANCE** Mixed fermentations using a controlled inoculation of Starmerella bacillaris and Saccharomyces cerevisiae starter cultures represent a feasible way to modulate wine composition that takes advantage of both the phenotypic specificities of the non-Saccharomyces strain and the ability of S. cerevisiae to complete wine fermentation. However, according to the composition of grape juices, the consumption by Starm. bacillaris of nutrients, in particular of nitrogen sources, during the first stages of the process may result in depletions that further limit the growth of S. cerevisiae and lead to stuck or sluggish fermentations. Consequently, understanding the preferences of non-Saccharomyces yeasts for the nitrogen sources available in grape must together with their phenotypic specificities is essential for an efficient implementation of sequential wine fermentations with Starm. bacillaris and S. cerevisiae species. The results of our study demonstrate a clear preference for ammonium compared to amino acids for the non-Saccharomyces species. This finding underlines the importance of nitrogen sources, which modulate the functional characteristics of inoculated yeast strains to better control the fermentation process and product quality.

## Abstract (precis)

Mixed fermentations using a controlled inoculation of Starmerella bacillaris and Saccharomyces cerevisiae starter cultures represent a feasible way to modulate wine composition that takes advantage of both the phenotypic specificities of the non-Saccharomyces strain and the ability of S. cerevisiae to complete wine fermentation. However, according to the composition of grape juices, the consumption by Starm. bacillaris of nutrients, in particular of nitrogen sources, during the first stages of the process may result in depletions that further limit the growth of S. cerevisiae and lead to stuck or sluggish fermentations. Consequently, understanding the preferences of non-Saccharomyces yeasts for the nitrogen sources available in grape must together with their phenotypic specificities is essential for an efficient implementation of sequential wine fermentations with Starm. bacillaris and S. cerevisiae species. The results of our study demonstrate a clear preference for ammonium compared to amino acids for the non-Saccharomyces species. This finding underlines the importance of nitrogen sources, which modulate the functional characteristics of inoculated yeast strains to better control the fermentation process and product quality.

## INTRODUCTION

Spontaneous wine fermentation is a complex process that is carried out by a succession of different yeast species and strains within a species that are resident populations of the winery or vineyard where grapes are grown (1). This fermentation practice allows wines to express the complexity of the vineyard microbiota and allows wine consumers to experience the nuances between different vineyards and vintages (2). The high degree of complexity that characterizes these wines is derived from an array of by-products produced from different native non-Saccharomyces and Saccharomyces cerevisiae yeasts (3). However, the evolution of agronomical practices together with climate variations increasing the average mean temperature in many wine regions has resulted in higher sugar contents in grapes and, consequently, in musts (4). In this context, there are growing problems of stuck or sluggish spontaneous fermentations (1). Furthermore, off-flavors, such as acetaldehyde, hydrogen sulfide, and volatile acidity, may be produced by the indigenous yeast species present in grape juices, most of which are regarded as spoilage microorganisms. As a consequence, producers are often forced to inoculate with selected yeasts to avoid uncomplete fermentations and production of undesirable aromas (2). Therefore, many winemakers inoculate musts with commercial S. cerevisiae strains to ensure a rapid increase in the S. cerevisiae cell number, to improve the fermentation rate, and to produce more predictable wines with established criteria (5).

Along with the addition of an S. cerevisiae strain, the use of mixed starter cultures with selected non-Saccharomyces and S. cerevisiae yeasts by simulating spontaneous fermentation can result in a greater complexity of wine and produce unusual aromas and flavors in ways not that cannot be attained with a pure starter culture of S. cerevisiae (6). The production of these complex aromas and flavors is mainly due to the ability of the nonconventional species to produce target metabolites or hydrolyze aromatic precursors (7). Despite these positive aspects, in recent years, concern regarding the use of sequential mixed-culture fermentations has been noted, because the initial growth of non-Saccharomyces yeasts may compete with S. cerevisiae for nutrients, limiting their subsequent growth and increasing the risk of sluggish or stuck fermentation (8).

The lack of nitrogen, in the form of ammonium and amino acids (yeast-assimilable nitrogen [YAN]), is often involved in problematic fermentation. This resource plays an important role in the fermentation progress, since it is essential for the growth and metabolic activity of yeasts. The nitrogen compounds are rapidly consumed by yeast cells during the first 24 to 36 h of fermentation to fill the biosynthetic pools of amino acids necessary for protein synthesis and growth (9). Moreover, the ability of strains to complete fermentation depends on the level of biomass production (10, 11), while nitrogen deficiency results in a lower biomass yield, which in turn decreases the fermentation rate and increases the time to complete fermentation. The absolute minimum concentration of nitrogen required for the completion of fermentation is very difficult to determine since the temperature, initial sugar concentration, and genetic background of the strain all modulate this parameter (12, 13). It is also important to note that not all nitrogen sources equally support yeast growth, because cells growing on ammonium, asparagine, or glutamine as the sole nitrogen source exhibit a 2-h generation time, while the generation time is increased by up to 4.5 h when yeasts are grown on tryptophan (14). Moreover, in the presence of amino acids and ammonium, wine yeasts sequentially take up nitrogen sources, and the order of assimilation is controlled by molecular mechanisms (15).

Among non-Saccharomyces yeasts, Starmerella bacillaris can occur at high numbers in grape musts (16). This species is known for its strong fructophilic character and its ability to produce low ethanol and high glycerol concentrations (17). Taking into consideration these characteristics, the coupling of Starm. bacillaris with selected S. cerevisiae strains has been proposed to improve wine. In particular, sequential fermentation with Starm. bacillaris and S. cerevisiae strains results in the reduction of ethanol in wines, which is a current challenge in the context of the constant increase in the sugar content of grape juice due to global climate change (18, 19). However, the achievement of fermentation and the final metabolite profiles are strain dependent and depend on having a fermentation environment, especially with regard to the delay between the Starm. bacillaris and S. cerevisiae inoculations (18, 20, 21). One of the most probable explanations for these observations that is worthwhile to investigate is a more pronounced exhaustion of nitrogen sources by Starm. bacillaris when S. cerevisiae is added, resulting in the limited implantation of this species.

In light of this evidence, a comprehensive exploration of the assimilation of complex nitrogen sources by both partners would be valuable to better exploit the potential of Starm. bacillaris during sequential fermentation with S. cerevisiae. To this end, the aim of this study was to evaluate nitrogen assimilation from complex nitrogen compounds (amino acids and ammonium) by Starm. bacillaris and S. cerevisiae during pure-culture fermentations, as well as to investigate the sequence of assimilation. The chemical compositions of wines were compared to each other to evaluate the impact of each species on the final product.

## RESULTS

### Growth and metabolite evolution during fermentation.

Starm. bacillaris and S. cerevisiae strains were grown in duplicate in SM200 synthetic medium with a high sugar concentration (229 g/liter) and 202 mg/liter of YAN, which consisted of a mixture of 19 amino acids and ammonium ions. The growth and the kinetics of metabolite formation from central carbon metabolism (CCM) were monitored according to the fermentation and profiles of the produced volatile compounds determined at the end of culturing.

**FIG 1:**
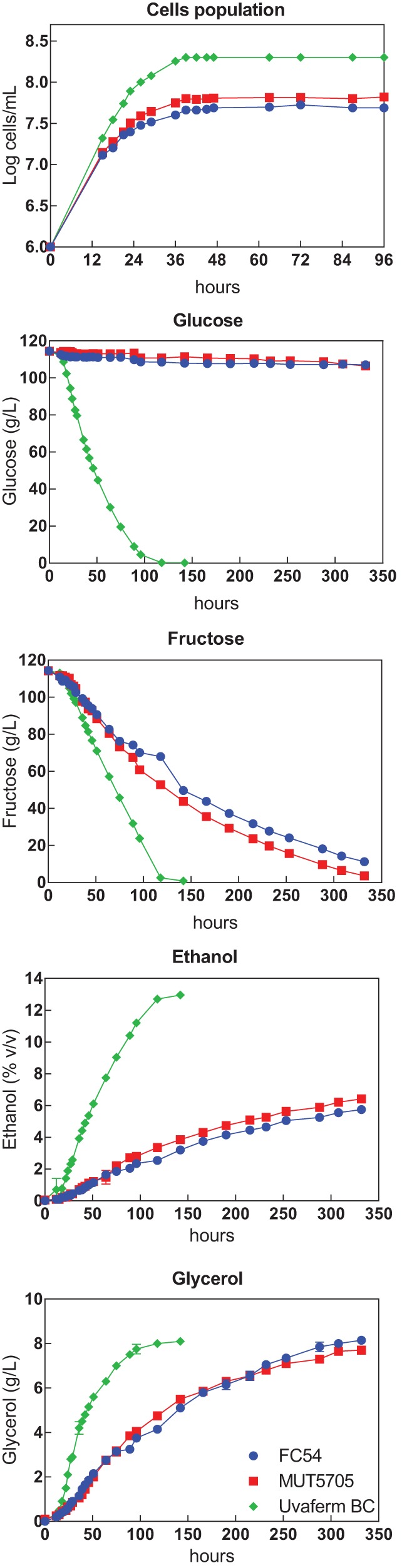
Growth dynamics and evolution of metabolites (glucose, fructose, ethanol, and glycerol) during pure-culture fermentations in SM200 inoculated with Saccharomyces cerevisiae and Starmerella bacillaris strains. Data are provided as the mean ± standard deviation of the results from two independent experiments. In general, the data for independent experiments were very similar, and a small standard deviation is therefore shown.

Both the growth and metabolite dynamics differed considerably between the two species, while the two Starm. bacillaris strains generally behaved uniformly (Fig. 1 and Table 1). S. cerevisiae Uvaferm BC reached a maximum population of 1.0 × 10^8^ cells/ml in 36 h and simultaneously consumed glucose and fructose, with a preference for glucose (118 versus 142 h for exhaustion, respectively). In contrast, a completely different picture emerged when Starm. bacillaris strains were used to ferment the must. Fermentation proceeded more slowly than with S. cerevisiae and stopped after 340 h. At this stage, almost all of the available fructose had been consumed (residual fructose, 3.7 to 11.3 g/liter), while glucose remained untouched (residual glucose, 106.5 to 107.1 g/liter). Furthermore, the two strains exhibited a similar growth dynamics pattern, reaching a cell population of about 7.6 × 10^7^ cells/ml in 48 h.

**TABLE 1:** Metabolites measured in wines produced by fermentation of synthetic must with S. cerevisiae and Starmerella bacillaris strains

Metabolite	Data by strain^*a*^			Significance
	Uvaferm BC	FC54	MUT5705	
Concn^*b*^				
Sugar consumption	228.5 ± 0.1 C	110.9 ± 0.1 A	119.1 ± 0.1 B	<0.001
Residual sugars	0.7 ± 0.1 A	118.4 ± 0.1 C	110.1 ± 0.1 B	<0.001
Glucose	0.1 ± 0.2 A	107.1 ± 0.1 B	106.5 ± 0.1 B	<0.001
Fructose	0.6 ± 0.1 A	11.3 ± 0.2 C	3.7 ± 0.4 B	<0.001
Biomass	3.89 ± 0.30 B	0.12 ± 0.20 A	0.10 ± 0.10 A	<0.001
Ethanol (% [vol/vol])	12.6 ± 0.3 C	5.8 ± 0.1 A	6.4 ± 0.1 B	<0.001
Glycerol	8.1 ± 0.2 B	8.2 ± 0.2 B	7.7 ± 0.1 A	<0.01
Acetic acid	0.64 ± 0.01 C	0.11 ± 0.01 A	0.21 ± 0.04 B	<0.001
Fumaric acid	0.13 ± 0.01 A	0.58 ± 0.02 C	0.59 ± 0.04 B	<0.001
Pyruvic acid	0.11 ± 0.05 A	0.87 ± 0.02 C	0.45 ± 0.01 B	<0.001
Succinic	0.80 ± 0.04 C	0.13 ± 0.02 A	0.24 ± 0.02 B	<0.001
α-Ketoglutaric acid	0.13 ± 0.02 A	0.37 ± 0.02 B	0.37 ± 0.03 B	<0.01
pH	3.31 ± 0.01 B	3.06 ± 0.01 A	3.11 ± 0.01 A	<0.001
Titratable acidity	12.17 ± 0.02 A	12.84 ± 0.01 B	13.11 ± 0.02 C	<0.001
Yields^*c*^				
Ethanol (% [vol/vol])	55.2 ± 0.1 B	52.5 ± 0.2 A	52.5 ± 0.2 A	<0.001
Glycerol (mg/g)	50.1 ± 0.1 A	76.5 ± 0.7 C	69.7 ± 0.7 B	<0.001
Acetic acid (mg/g)	3.9 ± 0.1 B	1.8 ± 0.1 A	1.5 ± 0.1 A	<0.001
Fumaric acid (mg/g)	0.6 ± 0.1 A	7.3 ± 0.4 C	4.9 ± 0.4 B	<0.001
Pyruvic acid (mg/g)	1.7 ± 0.1 A	6.8 ± 0.2 C	5.4 ± 0.1 B	<0.001
Succinic acid (mg/g)	3.4 ± 0.4 B	1.9 ± 0.1 A	2.1 ± 0.1 A	<0.05
α-Ketoglutaric acid (mg/g)	2.1 ± 0.1 A	3.4 ± 0.1 B	3.5 ± 0.1 B	<0.01

a The concentration of sugar at the beginning of experiment was 229.2 g/liter (114.7 g/liter glucose and 114.5 g/liter fructose). The values are from three independent experiments. Different uppercase letters within the same column indicate significant differences between pure- and mixed-culture fermentations (Tukey's b test, *P* < 0.05).

b Concentrations are in grams per liter unless otherwise indicated.

c Yields were calculated when both species consumed 100 g of sugars from the fermenting must.

The Starm. bacillaris strains were clearly differentiated from S. cerevisiae, as they produced large amounts of glycerol and organic acids and small amounts of ethanol and acetic acids (Table 1). Glycerol production was very similar for the two yeast species (7.7 to 8.2 g/liter) despite the differences in their sugar consumption levels. This similarity was due to the higher glycerol yields of Starm. bacillaris strains (69.7 to 76.5 mg/g) than those of S. cerevisiae (50 mg/g). Ethanol was significantly increased in wines fermented with S. cerevisiae, in accordance with the higher sugar consumption of this species. However, Starm. bacillaris strains displayed lower ethanol yields (a reduction of 2.7 mg/g) than Uvaferm BC (Table 1).

Large differences between S. cerevisiae and Starm. bacillaris strains were also found with regard to the yields of organic acids. First, the acetic acid yield of Starm. bacillaris strains (1.5 and 1.8 mg/g) was more than two times lower than that of S. cerevisiae (3.9 mg/g). Combined with the inefficient consumption of sugars by Starm. bacillaris, the reduced yield of acetic acid resulted in an important decrease in the formation of this compound during Starm. bacillaris fermentation (0.11 to 0.21 g/liter instead of 0.64 g/liter for S. cerevisiae). A similar pattern was observed in the production of succinic acid, with a lower production in Starm. bacillaris strains (0.13 to 0.24 g/liter) than in S. cerevisiae (0.80 g/liter) (Table 1). Conversely, the non-Saccharomyces strains exhibited higher yields of fumaric, pyruvic, and α-ketoglutaric acids than S. cerevisiae, resulting in increases of 77%, 77 to 87%, and 64% of their final concentrations, respectively. A significant decrease in pH with a parallel increase in titratable acidity of 0.67 to 0.94 g/liter was seen for wines produced using Starm. bacillaris strains. The differences were higher in wines produced from Starm. bacillaris MUT5705.

Higher alcohols were the most predominant volatile metabolite family in the produced wines, followed by acetate esters, ethyl esters, and volatile acids (Table 2). Substantial differences were found among the profiles of these aromas in wines produced by Starm. bacillaris strains from those produced by S. cerevisiae. Overall, the final concentrations of volatile metabolites, regardless of their family, were significantly lower in wines produced by Starm. bacillaris strains. In particular, the production of acetate and ethyl esters and of all of the volatile acids except butyric acid was strongly reduced in fermentation by Starm. bacillaris strains, while sugar consumption was only reduced by half. Decreases of 40-, 15-, and 7-fold in the formation of acetate esters, ethyl esters, and volatile acids by Starm. bacillaris were observed compared to those of S. cerevisiae Uvaferm BC, respectively. Conversely, the differences between strains with regard to the production of higher alcohols strongly depended on the nature of each individual compound. First, we found substantial decreases in the formation of methionol, 2-phenyl-1-ethanol, and 3-methyl-1-butanol by Starm. bacillaris FC54 and MUT5705, which only accounted for 14 to 19%, 12 to 15%, and 13 to 17% of those produced by S. cerevisiae Uvaferm BC, respectively. On the contrary, the production of propanol by Starm. bacillaris strains increased by 1.8-fold compared to that produced by S. cerevisiae Uvaferm BC. In the same way, a pronounced increase in the formation of 2-methyl-propanol was observed, while S. cerevisiae Uvaferm BC produced approximately 74 mg/liter 2-methyl-propanol and Starm. bacillaris FC54 and MUT5707 exhibited final production levels of 165 and 148 mg/liter 2-methyl-propanol, respectively. Finally, Starm. bacillaris strains displayed a low ability to synthetize both acetate and ethyl esters compared with S. cerevisiae strains, which could be explained by a low efficiency or a lack of acetyl transferases in this species.

**TABLE 2:** Concentrations of yeast volatile fermentation metabolites for wines produced by fermentation of synthetic must with S. cerevisiae and Starmerella bacillaris strains^*a*^

Compound	Concn (mean ± SD) (μg/liter)			Significance^*b*^
	Uvaferm BC	FC54	MUT5705	
Alcohols				
Propanol	4,133 ± 286 A	7,323 ± 533 B	7,476 ± 823 B	<0.001
Methionol	884 ± 50 B	124 ± 33 A	174 ± 17 A	<0.001
2-Methyl-1-propanol	73,987 ± 3,896 A	164,509 ± 23,550 B	147,844 ± 17,478 B	<0.01
2-Phenyl-1-ethanol	3,177 ± 298 B	381 ± 46 A	462 ± 131 A	<0.001
3-Methyl-1-butanol	308,333 ± 14,038 B	42,043 ± 9,252 A	52,091 ± 13,517 A	<0.001
∑ alcohols	390,516 ± 17,583 B	214,382 ± 20,197 A	208,049 ± 31,407 A	<0.001
Acetate esters				
Propyl-acetate	15.71 ± 1.13 B	0.96 ± 0.11 A	0.85 ± 0.01 A	<0.001
2-Methylpropyl acetate	35.68 ± 1.33 B	2.91 ± 0.04 A	3.14 ± 0.21 A	<0.001
2-Phenylethyl acetate	33.78 ± 1.20 B	0.18 ± 0.03 A	0.33 ± 0.44 A	<0.001
3-Methylbutyl acetate	154.72 ± 16.22 B	0.57 ± 0.11 A	0.35 ± 0.01 A	<0.001
∑ acetate esters	239.89 ± 19.24 B	4.62 ± 0.10 A	4.67 ± 0.20 A	<0.001
Ethyl esters				
Diethyl succinate	2.36 ± 0.51 B	1.14 ± 0.02 A	1.33 ± 0.11 A	<0.01
Ethyl butanoate	23.24 ± 0.52 B	1.96 ± 0.70 A	1.46 ± 0.18 A	<0.001
Ethyl decanoate	48.31 ± 4.21 B	1.37 ± 0.31 A	1.15 ± 0.12 A	<0.001
Ethyl dodecanoate	24.17 ± 7.70 B	2.89 ± 0.04 A	2.59 ± 0.53 A	<0.001
Ethyl hexanoate	51.2 ± 5.42 B	2.73 ± 0.61 A	3.49 ± 1.2 A	<0.001
Ethyl octanoate	88.77 ± 18 B	4.93 ± 0.82 A	5.41 ± 0.61 A	<0.001
Ethyl 2-methylbutanoate	0.13 ± 0.02 B	0.02 ± 0.03 A	0.06 ± 0.02 A	<0.001
∑ ethyl esters	238.18 ± 22.33 B	15.04 ± 1.90 A	15.48 ± 1.12 A	<0.001
Volatile acids				
Decanoic acid	8.58 ± 1.70 B	0.95 ± 0.51 A	1.42 ± 1.02 A	<0.001
Dodecanoic acid	2.68 ± 0.52 B	0.72 ± 0.60 A	0.44 ± 0.50 A	<0.01
Hexanoic acid	1.93 ± 0.64 B	0.26 ± 0.12 A	0.37 ± 0.12 A	<0.001
Isobutyric acid	0.95 ± 0.12	0.98 ± 0.80	1.03 ± 0.12	NS
Octanoic acid	44.71 ± 8.60 B	4.88 ± 0.50 A	4.96 ± 0.11 A	<0.001
Propanoic acid	8.37 ± 2.30 B	1.11 ± 0.10 A	1.15 ± 0.13 A	<0.001
Valeric acid	18.52 ± 1.43 B	2.22 ± 0.21 A	2.15 ± 0.24 A	<0.001
*∑* volatile acids	84.79 ± 14.59 B	11.10 ± 2.31 A	11.53 ± 1.51 A	<0.001

a Aroma compounds in wines from three independent experiments. Different letters within the same row indicate significant differences between the wines produced from S. cerevisiae and Starm. bacillaris strains (Tukey's b test; *P* < 0.05).

b NS, not significant.

### Nitrogen consumption. (i) Nitrogen uptake.

**FIG 2:**
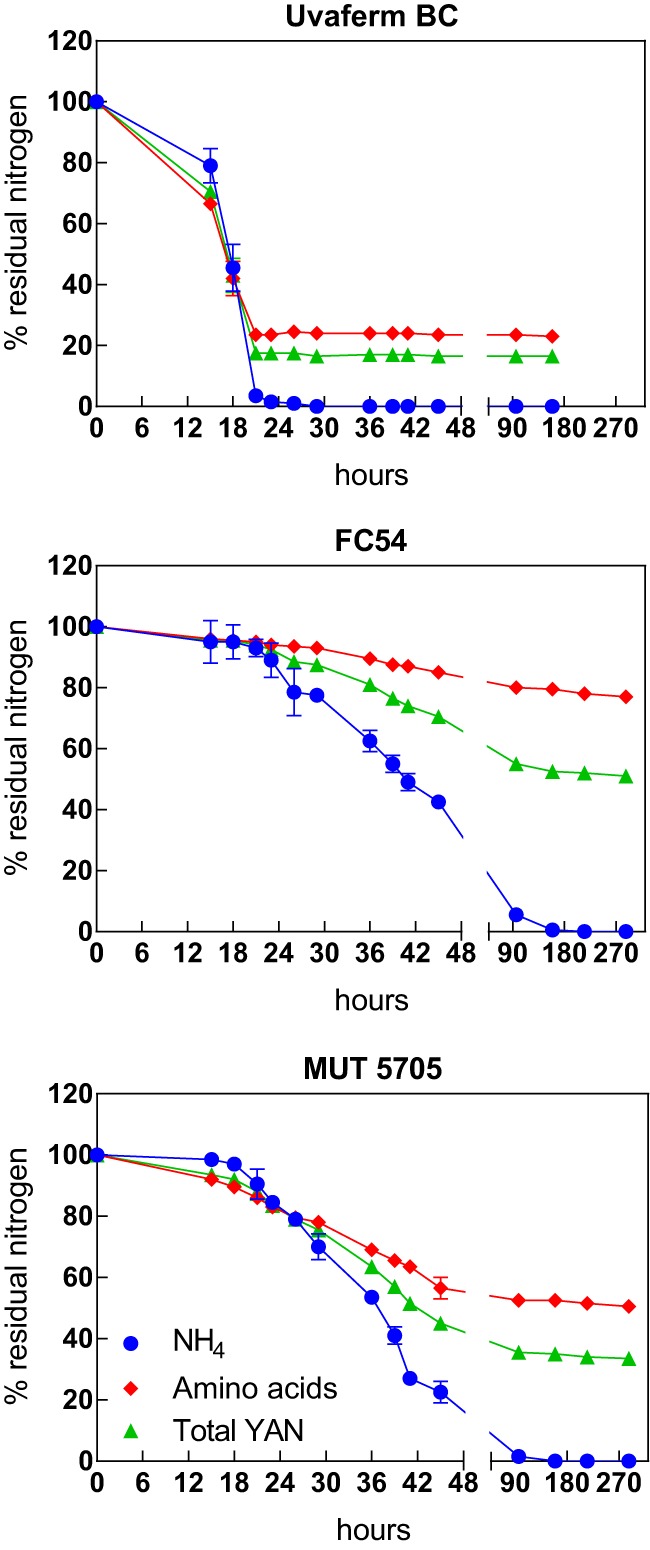
Consumption of yeast assimilable nitrogen (YAN), amino acids, and ammonium during pure-culture fermentations in SM200 inoculated with Saccharomyces cerevisiae and Starmerella bacillaris strains. The residual concentrations of each nitrogen compound are expressed as the percentages of the initial concentrations. Data are given as the mean ± standard deviation of the results from two independent experiments.

The profiles of total YAN, amino acids, and ammonium consumption by S. cerevisiae and Starm. bacillaris strains were monitored during the fermentation process (Fig. 2). Data for the amino acids alanine, glutamic acid, glycine, leucine, and valine were removed from the graphs due to the ability of Starm. bacillaris strains to produce these nitrogen compounds. Proline was also removed since none of the Starm. bacillaris or S. cerevisiae strains were able to consume this amino acid. All strains mainly consumed YAN during their growth phase, i.e., during the first 36 h and 48 h of fermentation for S. cerevisiae and Starm. bacillaris, respectively. However, the pattern of nitrogen consumption differed substantially between the two species. YAN was assimilated faster and at a greater quantity by S. cerevisiae Uvaferm BC. In particular, YAN was entirely exhausted after 30 h of Uvaferm BC fermentation, while the YAN concentration only decreased to a range of 58 (41%) to 111 (64%) mg N/liter when the Starm. bacillaris strains reached stationary phase. At this stage, both amino acids and ammonium remained at considerable amounts, independent of the Starm. bacillaris strain. However, ammonium continued to be consumed throughout the stationary phase and was fully depleted after 150 h of culture. On the contrary, Starm. bacillaris MUT5705 and FC54 consumed only 50% and 20% of the amino acids, respectively. Importantly, 50 to 80% of the available amino acids were still present in the medium at the end of the monitored period.

### (ii) Order of amino acid and ammonium uptake.

**FIG 3:**
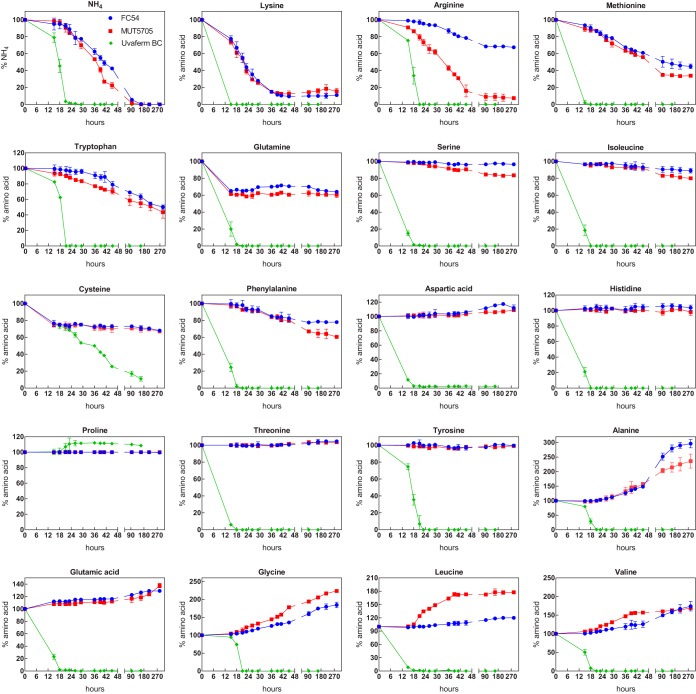
Consumption of individual amino acids (19) and ammonium during pure-culture fermentations inoculated with S. cerevisiae and Starm. bacillaris strains. The residual concentration of each nitrogen compound is expressed as the percentage of the initial concentrations. Data are given as the mean ± standard deviation of the results from two independent experiments.

To further investigate the variations between species with regard to their nutritional requirements for nitrogen, the consumption profiles of each N source during fermentation by the 3 strains were determined (Fig. 3). All of the strains displayed a sequential assimilation of the 20 nitrogen sources provided in the SM200 medium. S. cerevisiae Uvaferm BC was able to exhaust all of the amino acids provided in the synthetic grape juice except proline, according to the order of assimilation previously reported for 14 S. cerevisiae strains (15). In particular, prematurely consumed (Lys), early consumed (Asp, Thr, Glu, Leu, His, Met, Ile, Ser, Gln, and Phe), and late-consumed (ammonium, Val, Arg, Ala, Trp, Gly, and Tyr) nitrogen sources were able to be differentiated. Interestingly, the proline concentration at the end of the fermentation was greater than that initially present in the synthetic must.

Compared to S. cerevisiae, Starm. bacillaris showed very different patterns of assimilation of nitrogen sources (Fig. 3). The Starm. bacillaris strains exhibited the same consumption profile, except for arginine and leucine, and lacked the ability to efficiently take up a wide range of nitrogen compounds. In addition, the concentrations of some compounds surprisingly increased during fermentation by Starm. bacillaris strains. The possibility of releasing amino acids due to autolysis was discounted due to the limited loss of viability of the cells during the middle-end phases of fermentation (lower than 25%, Table S1). According to these profiles of consumption/production of amino acids, three clusters were identified. The first cluster included the nitrogen sources consumed by the Starm. bacillaris strains, ammonium, lysine, arginine, methionine, tryptophan, glutamine, serine, isoleucine, cysteine, and phenylalanine. Ammonium, lysine, methionine, tryptophan, and arginine (MUT5705) were efficiently (between 50 and 100%) taken up, with complete exhaustion only for ammonium, while the other compounds were consumed to only 30 to 40% of the amount provided in the medium. The second cluster consisted of aspartic acid, histidine, proline, serine, threonine, and tyrosine amino acids, for which the concentrations remained constant (or with low changes) throughout the fermentation. The last cluster contained alanine, glutamic acid, glycine, leucine (MUT5705), and valine. These amino acids were produced by Starm. bacillaris strains during the growth and stationary phases, with substantial increases in their concentrations at the end of the fermentation period. The most marked differences were observed for alanine (increase of approximately 170%), glycine (increase of approximately 100%), and valine (increase of approximately 70%). Moreover, the ability to produce substantial levels of leucine was strain dependent, as an 80% increase in the leucine content was observed throughout MUT5705 fermentation. In contrast, this increase was less than 20% for FC54.

### Role of the initial nitrogen concentration in nitrogen consumption.

**FIG 4:**
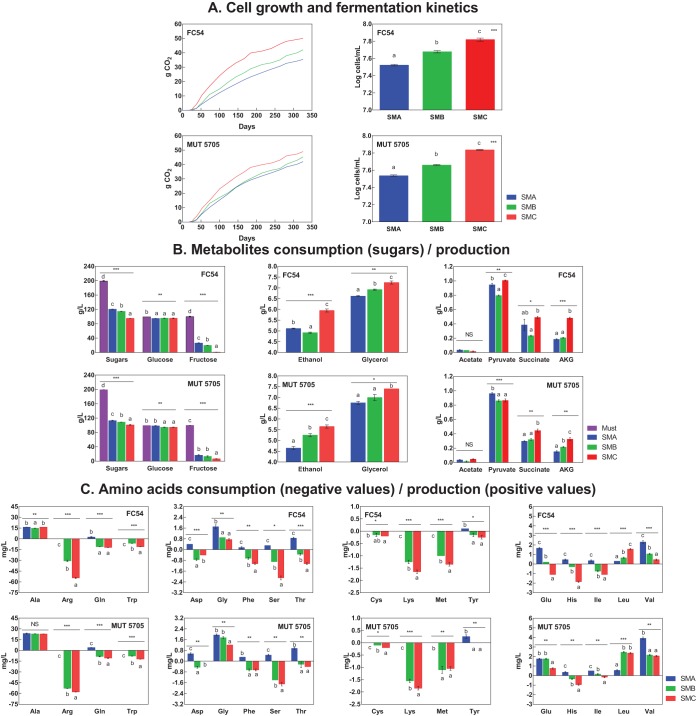
(A to C) Comparative analysis of the fermentation parameters obtained for both Starmerella bacillaris strains, with parameters related to growth (A), metabolite production (B), and amino acid consumption and production (C). Must A, 200 mg N/liter of NH_4_; must B, 178 and 22 mg N/liter of amino acids and NH_4_, respectively; and must C, 200 mg N/liter of amino acids. SMA, concentration of the metabolites at the end of the monitored period after fermentation of must A; SMB, concentration of the metabolites at the end of the monitored period after fermentation of must B; SMC, concentration of the metabolites at the end of monitored period after fermentation of must C. AKG, α-ketoglutaric acid. Different lowercase letters above the bars indicate significant differences among the synthetic musts used according to the Tukey's b test (*P* < 0.05). *, **, and *** indicate significance at *P* values of <0.05, <0.01, and <0.001, respectively.

The low consumption of amino acids by Starm. bacillaris compared with that of ammonium during wine fermentation appeared to be a specific feature of this species. To further investigate this particular phenotype, the FC54 and MUT5705 strains were grown on synthetic medium SM containing 200 mg N/liter of nitrogen as (i) the only ammonium source, (ii) a mixture of amino acids and ammonium, or (iii) a mixture of amino acids (Table 3). Interestingly, the growth and fermentation performances of both yeasts were significantly increased when the nitrogen resource was exclusively composed of amino acids (Fig. 4). In contrast, these characteristics were slightly decreased when ammonium was the sole nitrogen compound provided to support growth. Surprisingly, under these fermentation conditions, higher consumption of total nitrogen was observed than with fermentation in the presence of amino acids (110 to 134 mg N/liter versus 57 to 69 mg N/liter, respectively), even if less biomass was produced. In addition, most amino acids, apart from arginine, tryptophan, lysine, methionine, and cysteine, were released into the medium during growth. Furthermore, the two strains exhibited very similar profiles of amino acid production/consumption when amino acids were provided as the sole nitrogen source or in a mixture with ammonium. It is noteworthy that alanine, leucine, glycine, and valine were produced by Starm. bacillaris regardless of the nature of the N resources.

**TABLE 3:** Metabolites measured in wines produced by fermentation of synthetic musts with S. cerevisiae and Starm. bacillaris strains^*a*^

Parameter by metabolite	FC54 concn or yield (mean ± SD)			Significance	MUT5705 concn or yield (mean ± SD)			Significance
	SMA	SMB	SMC		SMA	SMB	SMC	
Concn (g/liter)								
Sugar consumption	78.8 ± 2.3 A	84.9 ± 5.9 B	103.8 ± 0.1 C	<0.01	86.9 ± 0.7 A	90.5 ± 2.8 B	98.8 ± 7.6 C	<0.001
Residual sugars	120.4 ± 2.3 C	114.2 ± 5.9 B	95.3 ± 0.1 A	<0.01	112.2 ± 0.7 C	108.7 ± 2.8 B	100.4 ± 7.6 A	<0.001
Glucose	94.6 ± 0.9	95.1 ± 2.2	94.6 ± 1.2	NS	97.1 ± 1.6 B	94.2 ± 2.6 A	94.2 ± 1.7 A	<0.05
Fructose	25.8 ± 1.4 C	19.2 ± 5.2 B	0.7 ± 1.0 A	<0.01	15.1 ± 2.4 B	14.5 ± 3.2 B	6.1 ± 5.9 A	<0.01
Ethanol (% [vol/vol])	5.1 ± 0.1 A	4.9 ± 0.3 A	5.9 ± 0.2 B	<0.001	4.6 ± 0.1 A	5.2 ± 0.2 B	5.7 ± 0.4 C	<0.01
Glycerol	6.6 ± 0.1 A	6.9 ± 0.1 B	7.3 ± 0.2 C	<0.01	6.8 ± 0.1 A	6.9 ± 0.1 A	7.4 ± 0.2 B	<0.05
Acetic acid	0.03 ± 0.01	0.03 ± 0.01	0.02 ± 0.03	NS	0.03 ± 0.01	0.01 ± 0.01	0.05 ± 0.04	NS
Fumaric acid	0.59 ± 0.06	0.55 ± 0.01	0.58 ± 0.01	NS	0.56 ± 0.03	0.58 ± 0.01	0.59 ± 0.01	NS
Pyruvic acid	0.93 ± 0.02 B	0.79 ± 0.03 A	1.00 ± 0.06 C	<0.01	0.95 ± 0.01 B	0.85 ± 0.01 A	0.85 ± 0.06 A	<0.001
Succinic	0.33 ± 0.08 AB	0.34 ± 0.02 A	0.48 ± 0.09 B	<0.05	0.30 ± 0.02 A	0.31 ± 0.02 A	0.43 ± 0.02 B	<0.01
α-Ketoglutaric acid	0.18 ± 0.01 A	0.21 ± 0.02 A	0.47 ± 0.04 B	<0.001	0.14 ± 0.01 A	0.21 ± 0.02 B	0.31 ± 0.09 C	<0.01
Yields								
Ethanol (% [vol/vol])	65.2 ± 1.1 B	58.4 ± 0.3 A	57.0 ± 1.5 A	<0.001	53.1 ± 0.3 A	57.6 ± 1.6 B	57.7 ± 0.1 B	<0.01
Glycerol (mg/g)	83.8 ± 1.2 B	84.5 ± 2.2 B	70.1 ± 0.2 A	<0.001	77.8 ± 0.1 B	75.9 ± 0.4 A	75.4 ± 0.1 A	<0.01
Acetic acid (mg/g)	0.2 ± 0.1	0.3 ± 0.1	0.2 ± 0.3	NS	0.4 ± 0.1	0.1 ± 0.1	0.5 ± 0.3	NS
Fumaric acid (mg/g)	7.4 ± 1.0 B	6.7 ± 0.1 B	5.5 ± 0.1 A	<0.05	6.4 ± 0.4	6.4 ± 0.3	6.0 ± 0.6	NS
Pyruvic acid (mg/g)	11.8 ± 0.6 B	9.6 ± 0.4 A	9.6 ± 0.6 A	<0.01	10.9 ± 0.2 B	9.5 ± 0.5 A	8.7 ± 1.3 A	<0.01
Succinic acid (mg/g)	4.2 ± 0.1 B	3.2 ± 0.2 A	4.6 ± 0.9 B	<0.05	3.4 ± 0.2 A	3.4 ± 0.5 A	4.4 ± 0.5 B	<0.05
α-Ketoglutaric acid (mg/g)	2.2 ± 0.2 A	2.6 ± 0.3 A	4.5 ± 0.4 B	<0.01	1.6 ± 0.1 A	2.2 ± 0.1 B	3.1 ± 0.7 C	<0.001

a The concentration of sugar at the beginning of experiment was 199.16 g/liter (99.23 g/liter glucose and 99.93 g/liter fructose). The values are the results from two independent experiments. SMA, 200.3 mg N/liter ammonium; SMB, 177.3 mg N/liter amino acids and 22.9 mg N/liter ammonium; SMC, 206.1 mg N/liter amino acid. Different uppercase letters within the same row indicate significant differences (A) among the strain FC54 and (B) among the strain MUT5705 (Tukey's b test; *P* < 0.05). NS, not significant.

## DISCUSSION

Currently, the use of non-Saccharomyces yeasts, such as Torulaspora delbrueckii, Lachancea thermotolerans, and Starm. bacillaris, in mixed-culture fermentations with selected S. cerevisiae strains is considered to be an up-to-date strategy that fulfills two main objectives (1, 6). First, due to the ability of non-Saccharomyces yeasts to produce high levels of glycerol, mannoproteins, organic acids that contribute to the total acidity, and volatile esters with pleasant notes, these yeasts provide a greater aromatic complexity to wines, increasing their quality (6, 7). Some non-Saccharomyces yeasts are also characterized by a limited production of acetic acid and ethanol during wine fermentation. Among these metabolites, ethanol reduction is of great interest as a consequence of global warming and consumer preference for well-structured and full-bodied wines produced from fully matured grapes (4). In this context, recent studies proposed the use of mixed-culture fermentations with selected Starm. bacillaris and S. cerevisiae strains to achieve this objective (18). However, attention must be paid to the nutrient concentration of the medium, since the initial growth of non-Saccharomyces in these fermentations can drastically reduce their availability and limit the subsequent growth of S. cerevisiae, thus increasing the risk of sluggish or stuck fermentations (8). Among nutrients, YAN plays a key role in regulating yeast growth, metabolism, and, as a result, the chemical and volatile compositions of the wines (22). Consequently, further knowledge of the nitrogen requirements of non-Saccharomyces species is needed to improve the use of these yeasts in mixed wine fermentation with S. cerevisiae.

### Specific features of Starmerella bacillaris related to the management of nitrogen.

In this study, focusing on the characterization of nitrogen metabolism by Starm. bacillaris in comparison with that by S. cerevisiae, we first noted substantial differences between the two species with regard to the amount and nature of nitrogen sources assimilated during fermentation. The main characteristic feature of Starm. bacillaris strains was their low assimilation of amino acids during wine fermentation, compared with ammonium, which was entirely consumed. Interestingly, the concentrations of several amino acids did not vary throughout fermentation, while some other amino acids were produced, such as alanine, glutamic acid, glycine, leucine (only for MUT5705), and valine.

Furthermore, differences in the earliest nitrogen sources consumed by the two species were observed. In particular, ammonium, tryptophan, and arginine were consumed in large part by Starm. bacillaris strains, but they were taken up only during the late stages of growth by S. cerevisiae. On the contrary, other amino acids that were more quickly consumed by S. cerevisiae, such as serine or threonine, were not assimilated by Starm. bacillaris strains.

Surprisingly, comparisons of fermentations in which nitrogen was only provided in an inorganic (ammonium) or an organic (mixture of amino acids) form revealed that organic N compounds supported Starm. bacillaris growth more efficiently than did ammonium. Overall, these observations led us to hypothesize that there are significant differences in the regulation of nitrogen uptake between Starm. bacillaris and S. cerevisiae. In S. cerevisiae, two regulatory mechanisms as well as the kinetic characteristics of transporters result in the sequential consumption of nitrogen compounds during the growth phase (15). High-affinity permeases under Ssy1p-Ptr3p-Ssy5 (SPS)-mediated control of transport led to the early consumption of amino acids, while the uptake of N compounds that were consumed late involved transporters that were under nitrogen catabolite repression (NCR) or were regulated by SPS low-affinity permeases (23, 24). The pattern of consumption of nitrogen sources by Starm. bacillaris reveals the strong inability of this species to take up most amino acids in the presence of ammonium. The molecular basis underlying the prevention of amino acid uptake by ammonium remains to be identified, but different explanations can be considered, such as less-efficient SPS-control methods of amino acid permeases or an inhibitory mechanism mediated by ammonium in Starm. bacillaris. Another explanation for the preferential use of ammonium by Starm. bacillaris is the use of an additional efficient system for ammonium uptake. In line with this assumption, Marini et al. (25) reported that ammonium can enter yeast cells via simple diffusion and using Mep-independent additional ammonium transport system when ammonium concentration drops. Finally, it is noteworthy that amino acids better sustain Starm. bacillaris growth than does ammonium, suggesting that the ability of yeasts to catabolize nitrogen sources to efficiently support growth is unconnected to their capacity for early consumption of these N molecules, as previously observed in S. cerevisiae (14, 15).

### Distinctive characteristics of Starmerella bacillaris in CCM.

**FIG 5:**
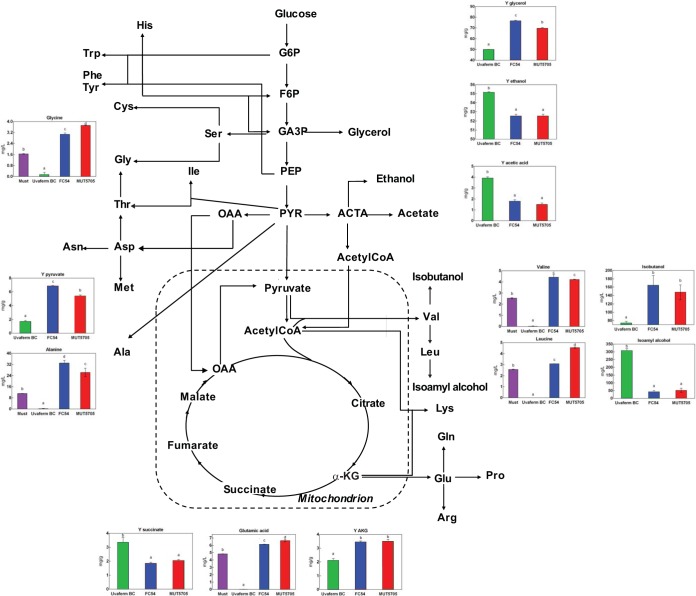
Intracellular carbon flux distribution of Saccharomyces cerevisiae and the Starmerella bacillaris strains. By-product yields (Y [milligrams per gram of sugar consumed]) and consumption/production of amino acids, isobutanol, and isoamyl alcohol for S. cerevisiae and the Starmerella bacillaris strains. Metabolites were measured after 150 and 300 h of fermentation for S. cerevisiae and the Starmerella bacillaris strains, respectively. Data are the mean ± standard deviation of the results from two independent experiments. The letters in each column indicate significant differences according to ANOVA and the Tukey's b test (*P* < 0.001). G6P, glucose 6-phosphate; F6P, fructose 6-phosphate; PEP, phosphoenolpyruvate; OAA, oxaloacetate; ACTA, acetaldehyde; PYR, pyroglutamic acid.

The comprehensive comparison of the consumption/production of amino acids, central carbon metabolism (CCM) metabolites, and volatile molecules between the two species, as summarized in Fig. 5, showed substantial differences in the flux partitioning of the central metabolic network, highlighting the specificities of Starm. bacillaris strains. The low production of ethanol and acetic acid by Starm. bacillaris strains compared to that of S. cerevisiae reveals the low activity of the acetaldehyde pathway in the non-Saccharomyces species. This decrease has large-scale effects on the metabolic fluxes, requiring increased production of glycerol to overcome the lower production of ethanol and to maintain the redox balance of cells (26, 27). Furthermore, there is a reorientation of fluxes around the pyruvic acid and glyceraldehyde-3-phosphate (GA3P) nodes that is in line with a reduced carbon channeling toward the acetaldehyde pathway in Starm. bacillaris, with increased production of pyruvate and amino acids and larger amounts of alcohols derived from this intermediate (alanine, leucine, valine, and isobutanol), as well as metabolites from GA3P (glycine and glycerol).

Surprisingly, though isoamyl alcohol and isobutanol are derived from the same metabolic pathway (28), only the production of isobutanol was increased. In contrast, the formation of isoamyl alcohol was drastically decreased in the Starm. bacillaris strains. Different variations in the production of these higher alcohols by S. cerevisiae in response to environmental modifications have been previously reported (28–30). These various responses according to the nature of the higher alcohol have been shown to be due to changes in acetyl-coenzyme (acetyl-CoA) availability, which is required for the conversion of α-ketobutyrate (KIB), the precursor of isobutanol, into α-ketoisovalerate (KIV), the precursor of isoamyl alcohol (31). Thus, the strongly reduced formation of isoamyl alcohol by Starm. bacillaris species is likely due to a decrease in acetyl-CoA availability, which could be, in turn, explained by the low flux through the acetaldehyde pathway. In agreement with a strong limitation of the intracellular pool of acetyl-CoA in non-Saccharomyces species, the formation of all of the volatile esters and acids by Starm. bacillaris, which are acetyl-CoA dependent, is considerably low compared to that by S. cerevisiae.

During fermentation, the tricarboxylic acid (TCA) pathway operates as two branches, and the main role of the oxidative route is to provide precursors for anabolism (32, 33). Compared to those of S. cerevisiae, the production yields of α-ketoglutaric acid and glutamic acid of the Starm. bacillaris were increased by 0.0015 mg/g and 1.0 to 1.5 mg/g, respectively. In contrast, the formation of succinic acid fell by 0.0015 mg/g. These variations emphasize a redistribution of fluxes from the TCA intermediate α-ketoglutaric acid toward the formation of glutamate at the expense of succinate in Starm. bacillaris strains. This redistribution may either reflect specific management of the nitrogen resource by this species or may instead be explained by the low capacity of Starm. bacillaris strains to convert α-ketoglutaric acid into succinic acid.

In conclusion, this study highlighted the specific phenotypic features of Starm. bacillaris strains during wine fermentation, in addition to their extremely fructophilic character (19). In particular, compared with S. cerevisiae, this non-Saccharomyces yeast exhibits low activity through the acetaldehyde pathway, which triggers an important redistribution of fluxes through the central carbon metabolic network. Furthermore, the two species differ with regard to their pattern of consumption of the wine complex nitrogen resource and their requirements for nitrogen nutrients. From an industrial perspective, these findings provide new relevant prospects in the field of oenology to improve the quality of wines. Thus, in line with the metabolic reorientations around the pyruvate and GA3P nodes of Starm. bacillaris, the use of this species in coinoculation or sequential inoculation with S. cerevisiae may allow a decrease in the ethanol and acetate contents of wines, with increased production of glycerol, which may also address a key issue of the winemaking industry in the context of global warming (32, 33). A main challenge for the future will be to further decipher the carbon flux distribution in Starm. bacillaris cells underlying the phenotypes obtained. Otherwise, the advantages of using Starm. bacillaris are the limited nitrogen requirements of the non-Saccharomyces yeast and its ability to excrete some amino acids, in particular, branched amino acids, during sequential fermentation with S. cerevisiae. S. cerevisiae may use the released amino acids to sustain its growth or to produce volatile molecules of interest derived from branched N compounds.

## MATERIALS AND METHODS

### Yeast strains.

Two Starm. bacillaris strains and one S. cerevisiae strain were used in this study. The Starm. bacillaris strains were FC54 and MUT705 from the yeast culture collection of DISAFA (Department of Agricultural, Forest and Food Sciences, University of Torino, Italy) and MUT (Mycotheca Universitatis Taurinensis, DBIOS, University of Torino, Italy), respectively. The commercial S. cerevisiae strain Uvaferm BC (Lallemand, Inc., Montreal, Canada) was used as a reference strain.

### Inoculation procedure.

For each strain, an aliquot of frozen cells (maintained at −80°C) was propagated at 28°C in YPD broth (1% yeast extract, 2% peptone, and 2% glucose; Oxoid, Paris, France) and streaked onto YPD agar plates to obtain single colonies 72 h before fermentation. Afterwards, one fresh colony was selected to inoculate 10 ml of YPD medium in a 50-ml Erlenmeyer flask at 28°C with continuous shaking (150 rpm). After 24 h of incubation, an aliquot of culture was used to inoculate 10 ml of synthetic or natural grape must at an initial cell population of 1.0 × 10^6^ cells/ml. The inoculum was grown under the same conditions for another 24 h.

### Fermentation media.

Fermentations were performed in synthetic medium called SM200, which simulates standard grape juice at pH 3.3. The medium was prepared using the protocols described by Bely et al. (34), with the following modifications regarding the sugars and YAN concentrations: 114.7 g/liter glucose, 114.5 g/liter fructose, and 202 ± 5.4 mg/liter YAN as a mixture of 19 amino acids (132.9 ± 3.9 mg N/liter) and ammonium salt (69.1 ± 1.5 mg N/liter). Fermentations were performed in duplicate in 1.2-liter glass fermenters containing 1.1 liters of synthetic medium that was previously flash-pasteurized and inoculated with 1.0 × 10^6^ cells/ml using the above-mentioned inoculum. Fermenters were equipped with fermentation air-locks to maintain semianaerobic conditions and incubated at 25°C with continuous magnetic stirring (300 rpm). Fermentations were stopped when the weight loss remained constant for two consecutive days. The reference medium (SM200) was supplied with various mixtures of amino acids and ammonium to form 3 different musts (Table 4). The composition of the musts was as follows (in milligrams of N per liter): SMA, 200.3 ammonium; SM200B, 177.3 amino acids and 22.9 ammonium; and SM200C, 206.1 amino acids. These fermentations were conducted in duplicate in 330-ml glass fermenters under the above-mentioned fermentation conditions.

**TABLE 4:** Initial and final concentrations of ammonium and amino acids in the synthetic musts used in this study

Nitrogen compound	Concn (mg N/liter)^*a*^								
	SMA			SMB			SMC		
	Must	FC54	MUT5705	Must	FC54	MUT5705	Must	FC54	MUT5705
Amino acids									
Alanine	ND	16.3 ± 0.2	23.1 ± 0.1	12.2 ± 0.1	26.9 ± 0.2	35.4 ± 0.2	13.5 ± 0.1	29.8 ± 0.2	36.3 ± 0.4
Arginine	ND	ND	ND	62.6 ± 0.2	31.1 ± 1.1	9.9 ± 0.1	73.4 ± 0.1	19.4 ± 0.8	15.7 ± 0.1
Aspartic acid	ND	0.4 ± 0.2	0.5 ± 0.1	2.9 ± 0.1	2.2 ± 0.1	2.4 ± 0.1	2.7 ± 0.2	2.3 ± 0.1	2.7 ± 0.2
Cysteine	ND	ND	ND	0.5 ± 0.2	0.4 ± 0.2	0.4 ± 0.1	0.6 ± 0.1	0.4 ± 0.2	0.4 ± 0.1
Glutamine	ND	3.0 ± 0.2	4.1 ± 0.2	15.3 ± 0.1	4.4 ± 0.2	7.3 ± 0.2	16.9 ± 0.1	5.1 ± 0.1	6.5 ± 0.7
Glutamic acid	ND	1.6 ± 0.1	1.7 ± 0.1	5.9 ± 0.1	6.1 ± 0.1	7.6 ± 0.5	6.9 ± 0.1	5.8 ± 0.2	7.6 ± 0.1
Glycine	ND	1.6 ± 0.2	1.9 ± 0.1	1.8 ± 0.1	2.7 ± 0.1	3.5 ± 0.1	2.1 ± 0.1	2.8 ± 0.1	3.3 ± 0.1
Histidine	ND	0.4 ± 0.3	0.3 ± 0.1	4.4 ± 0.2	4.1 ± 0.2	4.1 ± 0.1	5.3 ± 0.2	3.5 ± 0.2	4.4 ± 0.3
Isoleucine	ND	0.3 ± 0.1	0.5 ± 0.2	1.8 ± 0.2	1.1 ± 0.1	1.9 ± 0.1	2.0 ± 0.1	0.9 ± 0.1	1.9 ± 0.2
Leucine	ND	0.3 ± 0.1	0.5 ± 0.2	2.8 ± 0.1	3.4 ± 0.1	5.3 ± 0.2	3.2 ± 0.1	4.7 ± 0.1	5.5 ± 0.1
Lysine	ND	ND	ND	1.7 ± 0.1	0.4 ± 0.2	0.2 ± 0.1	1.9 ± 0.1	0.3 ± 0.2	0.1 ± 0.0
Methionine	ND	ND	ND	1.4 ± 0.1	0.4 ± 0.1	0.4 ± 0.2	1.6 ± 0.1	0.3 ± 0.1	0.5 ± 0.0
Phenylalanine	ND	0.2 ± 0.2	0.3 ± 0.2	1.7 ± 0.1	1.1 ± 0.1	1.1 ± 0.1	1.9 ± 0.2	0.9 ± 0.2	1.3 ± 0.2
Proline	ND	1.2 ± 0.1	1.1 ± 0.2	36.8 ± 0	36.3 ± 0.1	36.8 ± 0.2	41.6 ± 0.1	41.9 ± 0.1	42.6 ± 0.1
Serine	ND	0.3 ± 0.1	0.4 ± 0.1	5.5 ± 0.1	4.6 ± 0.4	4.1 ± 0.1	6.2 ± 0.1	4.2 ± 0.2	4.6 ± 0.2
Threonine	ND	0.8 ± 0.1	0.9 ± 0.1	4.7 ± 0.1	4.4 ± 0.1	4.3 ± 0.1	5.3 ± 0.1	4.3 ± 0.1	4.9 ± 0.2
Tryptophan	ND	ND	ND	11.8 ± 0.1	5.5 ± 0.1	4.1 ± 0.2	17.4 ± 0.2	6.6 ± 0.2	5.2 ± 0.2
Tyrosine	ND	0.1 ± 0.0	0.2 ± 0.2	0.7 ± 0.2	0.6 ± 0.1	0.7 ± 0.1	0.9 ± 0.1	0.7 ± 0.2	0.9 ± 0.1
Valine	ND	2.2 ± 0.1	3.8 ± 0.1	2.8 ± 0.3	3.8 ± 0.1	4.9 ± 0.1	3.2 ± 0.1	3.6 ± 0.1	5.2 ± 0.1
NH_4_	200.3 ± 1.3	30.8 ± 1.2	25.9 ± 4.3	22.9 ± 0.1	ND	ND	ND	ND	ND
Total amino acids	ND	28.4 ± 0.4	39.1 ± 0.1	177.3 ± 0.6	139.5 ± 1.7	134 ± 0.4	206.1 ± 0.2	137.3 ± 1.3	149.3 ± 0.8
Total YAN	200.3 ± 1.3	90.1 ± 2.1	65.8 ± 4.4	200.2 ± 0.7	139.5 ± 1.7	134 ± 0.4	206.1 ± 0.2	137.3 ± 1.3	149.3 ± 0.8

a SMA, 200.3 mg N/liter ammonium; SMB, 177.3 mg N/liter amino acids and 22.9 mg N/liter ammonium; SMC, 206.1 mg N/liter amino acids. ND, not detected.

### Analytical methods.

Cell densities were monitored every 3 h from 12 to 48 h and then once a day from 48 to 96 h of fermentation by counting cells using an electronic particle counter (Multisizer 3 Coulter Counter; Beckman Coulter) after sonication to separate aggregated cells. Cell viability during the middle-end phases of fermentation was determined with an epifluorescent method using a C6 cytometer (Accuri, BD Biosciences, San Jose, CA), as described by Delobel et al. (35). Briefly, cells were stained with propidium iodide (PI), an indicator of cell viability that works due its inability to penetrate intact cell membranes. Viability was determined as the percentage of intact and fragile cells among all cells. Each sample was analyzed using three biological replicates.

The total YAN concentration was determined according to the sum of organic (amino acids) and inorganic nitrogen (ammonium). Before the quantification of free amino acids, molecules with high molecular weights were removed from the samples by the addition of 200 μl of a sulfosalicylic acid solution (25% [wt/vol]) to 800 μl of sample, followed by incubation at 4°C for 1 h. After centrifugation at 14,000 rpm for 10 min, the samples were filtered through a 0.22-μm-pore-size Millipore nitrocellulose membrane. Amino acid identification and quantification were performed by liquid chromatography with a Biochrom 30 amino acid analyzer (Biochrom Ltd., Cambridge, UK) under the chromatographic conditions reported by Crépin et al. (15). The ammonium concentration was assayed spectrophotometrically using an enzymatic kit (R-Biopharm AG, Darmstadt, Germany), according to the manufacturer's instructions.

The extracellular sugar, ethanol, glycerol, and organic acid (acetic, fumaric, pyruvic, α-ketoglutaric, and succinic acids) concentrations in the samples were determined by high-performance liquid chromatography (HPLC; HPLC 1290 Infinity; Agilent Technologies, Santa Clara, CA, USA) using an HPX-87H ion exclusion column (Bio-Rad). The column was eluted with 0.005 N H_2_SO_4_ at a flow rate of 0.6 ml/min. The organic acid concentrations were determined with a UV meter at 210 nm, while the concentrations of the other compounds were determined with a refractive index detector (32). A total of 23 volatile metabolites were identified in the fermented wines, and these compounds included 5 higher alcohols, 4 acetate esters, 7 ethyl esters, and 7 volatile acids. Analyses were performed by gas chromatography-mass spectrometry according to the protocols reported by Rollero et al. (36). The accuracy of the quantification of the metabolites was achieved with the use of poly(deuterated) internal standards for stable isotope dilution analysis (37).

### Statistical analyses.

Differences were established using one-way analysis of variance (ANOVA), followed by the software IBM SPSS Statistics package (version 19.0; IBM Corp., Armonk, NY, USA). ANOVA was coupled with the Tukey's b post hoc test when *P* values were lower than 0.05 to evaluate significant differences.

## Supplementary Material

Supplemental material
