# Supplementary material for: Specific Phenotypic Traits of Starmerella bacillaris Related to Nitrogen Source Consumption and Central Carbon Metabolite Production during Wine Fermentation
Source: Appl Environ Microbiol. 2018 Aug 1;84(16):e00797-18. doi: 10.1128/AEM.00797-18 (PMC6070767; doi:10.1128/AEM.00797-18)
Supplement: Supplemental material [file supp_84_16_e00797-18__index.html]

Supplemental material 

# Specific Phenotypic Traits of Starmerella bacillaris Related to Nitrogen Source Consumption and Central Carbon Metabolite Production during Wine Fermentation

## Supplemental material

- Supplemental file 1 -

  Cell viability during the middle-end phases of fermentation (Table S1).

  PDF, 8.1K
