# Supplementary material for: Specific Phenotypic Traits of Starmerella bacillaris Related to Nitrogen Source Consumption and Central Carbon Metabolite Production during Wine Fermentation
Source: Appl Environ Microbiol. 2018 Aug 1;84(16):e00797-18. doi: 10.1128/AEM.00797-18 (PMC6070767; doi:10.1128/AEM.00797-18)
Supplement: Supplemental material [file AEM.00797-18_zam016188662s1.pdf]

Table S1

Cells viability during the middle-end phases of fermentation

| Strains | Intact cell population | Dead cell population |
|---|---|---|
| FC54 | 75.6 % | 18.2% |
| MUT 5705 | 74.9 % | 19.45 |
